# Aquaporin-4 and brain edema

**DOI:** 10.1007/s00467-006-0411-0

**Published:** 2007-06-01

**Authors:** Marios C. Papadopoulos, Alan S. Verkman

**Affiliations:** 1 Departments of Medicine and Physiology, University of California, San Francisco, CA 94143-0521 USA; 2 Academic Neurosurgery Unit, St. George’s University of London, Cranmer Terrace, Tooting, London, SW17 0RE UK; 3 Cardiovascular Research Institute, University of California, 1246 Health Sciences East Tower, Box 0521, San Francisco, CA 94143-0521 USA

**Keywords:** AQP4, Brain swelling, Water channel, Hydrocephalus, Hyponatremia

## Abstract

Aquaporin-4 (AQP4) is a water-channel protein expressed strongly in the brain, predominantly in astrocyte foot processes at the borders between the brain parenchyma and major fluid compartments, including cerebrospinal fluid (CSF) and blood. This distribution suggests that AQP4 controls water fluxes into and out of the brain parenchyma. Experiments using AQP4-null mice provide strong evidence for AQP4 involvement in cerebral water balance. AQP4-null mice are protected from cellular (cytotoxic) brain edema produced by water intoxication, brain ischemia, or meningitis. However, AQP4 deletion aggravates vasogenic (fluid leak) brain edema produced by tumor, cortical freeze, intraparenchymal fluid infusion, or brain abscess. In cytotoxic edema, AQP4 deletion slows the rate of water entry into brain, whereas in vasogenic edema, AQP4 deletion reduces the rate of water outflow from brain parenchyma. AQP4 deletion also worsens obstructive hydrocephalus. Recently, AQP4 was also found to play a major role in processes unrelated to brain edema, including astrocyte migration and neuronal excitability. These findings suggest that modulation of AQP4 expression or function may be beneficial in several cerebral disorders, including hyponatremic brain edema, hydrocephalus, stroke, tumor, infection, epilepsy, and traumatic brain injury.

## Introduction

The aquaporins (AQPs) are a family of water-channel proteins. The first AQP was identified in red blood cells in 1991 and called AQP1 [1]. Over the last 15 years, at least 13 AQPs have been discovered in mammals. The aquaporins are tetramers, each monomer having its own water pore. AQPs primarily transport water, except for AQP3, AQP7, and AQP9, the aquaglyceroporins, which also transport glycerol and various small polar molecules. Here we review the mechanisms of brain edema formation and absorption and discuss newly discovered roles of AQP4.

### Aquaporins in nonbrain tissues

**Fig. 1:**
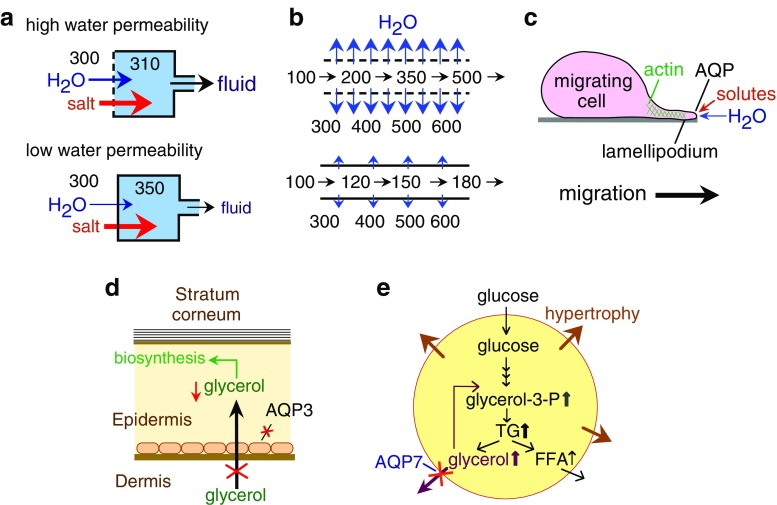
Mechanisms of aquaporin (AQP) function outside the brain. **a** Reduced water permeability in glandular epithelium impairs active, near-isosmolar fluid transport by slowing osmotic water transport into the acinar lumen, producing hypertonic secretion. **b** Reduced transepithelial water permeability in kidney collecting duct impairs urinary concentrating ability by preventing osmotic equilibration of luminal fluid. **c** AQP-facilitated water entry into protruding lamellipodia, accounting for AQP-dependent cell migration. **d** Reduced steady-state glycerol content in epidermis and stratum corneum following AQP3 deletion, accounting for reduced skin hydration in AQP3 deficiency. **e** Impaired AQP7-dependent glycerol escape from adipocytes, resulting in intracellular glycerol accumulation and increased triglyceride content accounting for progressive adipocyte hypertrophy in AQP7 deficiency

Analysis of transgenic mice lacking specific AQPs has provided insight into the physiological roles of AQPs (reviewed in [2]). Mice lacking several AQPs (AQPs 1–4) manifest a defect in urinary concentrating ability [3]. Near-isosmolar fluid secretion is impaired in salivary and airway submucosal gland in AQP5 deficiency. The general paradigm from these findings is that high transepithelial water permeability permits rapid water transport in response to active transepithelial salt transport. AQP deletion impairs osmotic equilibration, resulting in reduced volume of relatively hypertonic fluid secretion (Fig. 1a). A second mechanism for involvement of AQPs in mammalian physiology is facilitation of passive, osmotically driven water transport, as in osmotic water extraction in kidney collecting duct (Fig. 1b). As discussed below with regard to astrocytes, a newly discovered role of AQPs is cell migration facilitation. We propose that the mechanism for this facilitation is that actin cleavage and ion uptake at the tip of a lamellipodium create local osmotic gradients that drive water influx (Fig. 1c).

The aquaglyceroporins have unique biological roles related to their glycerol transport function. AQP3-facilitated glycerol transport in skin is an important determinant of epidermal and stratum corneum hydration. AQP3 is expressed strongly in the basal layer of keratinocytes in mammalian skin. Mice lacking AQP3 have reduced stratum corneum hydration and skin elasticity and impaired stratum corneum biosynthesis and wound healing. The mechanism responsible for the skin phenotype in AQP3 deficiency involves reduced epidermal-cell skin glycerol permeability, resulting in reduced glycerol content in the stratum corneum and epidermis (Fig. 1d). Another aquaglyceroporin, AQP7, is expressed in the plasma membrane of adipocytes. AQP7-null mice have a greater fat mass than do wild-type mice as they age, with remarkable adipocyte hypertrophy and accumulation of glycerol and triglycerides. Hypertrophy of AQP7-deficient adipocytes probably results from reduced plasma membrane glycerol permeability and consequent increased glycerol accumulation and triglyceride biosynthesis (Fig. 1e).

### Aquaporins in brain

AQP4 is the primary water channel found in the brain, but AQP1 and AQP9 have also been reported. In this review, we concentrated on AQP4, which is much more abundantly expressed in brain than is AQP1 or AQP9. AQP1 is expressed in the choroid plexus and plays a role in cerebrospinal fluid (CSF) formation [4]. AQP9 protein has been detected weakly by antibody staining by some groups in some astrocytes processes at the glia limitans [5] and in ependymal cells and tanycytes [6].

### AQP4 expression

AQP4 expression, studied using immunohistochemistry and immunoelectron microscopy, was found in glia only. AQP4 is expressed in astrocyte foot processes surrounding capillaries, astrocyte processes comprising the glial limiting membrane, in ependymal cells, and in subependymal astrocytes [7, 8]. Although AQP4 is also expressed in the supraoptic and suprachiasmatic nuclei of the hypothalamus, AQP4-null mice do not have hypothalamic disturbances [9]. The pattern of AQP4 protein expression, predominantly at the borders between the brain parenchyma and major fluid compartments, suggests involvement of AQP4 in water movement into and out of the brain parenchyma.

The mechanisms described below are based on experiments involving adult mice. Because the pattern of expression of aquaporins in human brain is similar to that of the mouse brain, these mechanisms probably apply to humans, also. However, little is known about edema formation and absorption in neonates. Neonates have less prominent AQP4 expression in the brain [10], immature blood–brain [11, 12] and blood–CSF [13] barriers, and increased extracellular space volume [14]. These differences between the neonate and adult brain may be important, and therefore, mechanisms of edema formation and elimination may be different.

## Brain edema

Brain edema is brain swelling that occurs due to the accumulation of excess water in the brain parenchyma [15, 16]. Brain edema is associated with several brain pathologies, such as hydrocephalus, traumatic brain injury, stroke, and brain tumors, as well as extracranial pathologies that affect the brain secondarily, including hyponatremia, organ failure (liver, kidney), and sepsis. Brain edema is also seen in globally important systemic infections that primarily involve the brain, such as childhood cerebral malaria and meningitis.

### Brain water homeostasis

In the normal adult brain, water is distributed between several compartments, including CSF (CSF, ~75–100 ml), blood (~75–100 ml) and intracellular (1,100–1,300 ml) and interstitial (~100–150 ml) brain parenchyma. Water moves between the different compartments in response to osmotic gradients and hydrostatic pressure differences. Because the brain is enclosed in a rigid skull, brain swelling produces displacement of water from low-pressure compartments, including CSF and venous blood (~10 mmHg), into peripheral blood. Once the low-pressure reserve is exhausted, intracranial pressure progressively rises; the sulci become effaced on computed tomography (CT) and magnetic resonance image (MRI) scans, then the third ventricle becomes invisible, and finally the basal cisterns disappear due to brain-stem herniation. High intracranial pressure can therefore cause brain ischemia, herniation, and death. This scenario, however, does not apply to young children (less than a year old) in whom the open fontanelles can accommodate, to some extent, the swollen brain. In this situation, the fontanelles bulge and the head circumference increases, minimizing the rise in intracranial pressure. However, even the open fontanelles cannot compensate for rapid brain swelling, and therefore, young children are not fully resistant to the development of high intracranial pressure and brain herniation.

### Brain edema management

Brain edema management includes sedation and avoidance of hypercapnia to prevent intracranial pressure elevation, administration of intravenous hyperosmolar solutions such as mannitol and hypertonic saline, corticosteroids for brain tumors, surgical resection of the causative lesion, and in extreme cases, decompressive craniectomy. For critically ill patients, invasive monitoring of intracranial pressure and cerebral perfusion pressure is done to optimize therapy. However, many of these therapies to reduce brain swelling were introduced early in the early/mid twentieth century, and their efficacy is limited [17, 18]. The paucity of effective drugs to be used in brain edema reflects, in part, the incomplete understanding of cellular mechanisms involved in brain edema formation and resolution.

### Edema formation mechanisms

A logical framework for understanding the cellular mechanisms of brain edema was first suggested by Igor Klatzo in the 1960s, when he classified edema into cytotoxic and vasogenic types; a third type, termed hydrocephalic edema, has subsequently been described [15]. Common causes of brain edema are summarized in the Table 1.

**Table 1:** Some causes of brain edema

Cytotoxic	Vasogenic	Hydrocephalic
Vascular	Brain tumor	Obstructive
Early hypoxia	Supratentorial	Tumor
Early ischemia	Infratentorial	Aqueduct stenosis
		Chiari malformation
		Dandy-Walker
Infection	Infection	Communicating
Cerebral malaria	Meningitis	Meningitis
Meningitis	Abscess	Meningitis
Subdural	Empyema	Subarachnoid hemorrhage
		Intraventricular hemorrhage
Metabolic		
Hyponatremia	Traumatic	
Hyperammonemia		
Diabetic ketoacidosis	Vascular	
Hyperbilirubinemia	Late hypoxia	
Uremic	Late ischemia	
Traumatic		

*Cytotoxic edema*, as seen in hyponatremia and early cerebral ischemia, is intracellular accumulation of water due to energy failure and inability of cells to regulate their volume [16]. This results in a shift of water from the interstitial into the intracellular compartment and a net uptake of water from the blood compartment into the brain parenchyma. Astrocytes are the main cell type that swell in cytotoxic brain edema, especially the pericapillary foot processes [16], which are the predominant sites of AQP4 expression in the brain [8, 19]. Astrocyte swelling may be an important early event predisposing the brain to further damage. Because cytotoxic edema affects all cells, both gray and white matter swell, resulting in loss of the clear margin between gray and white matter on CT and MRI scans. Because the blood–brain barrier is intact, excess brain water is not accompanied by protein, and there is no brain enhancement on CT or MRI scans after intravenous contrast administration.

*Vasogenic edema*, of which brain tumor and brain abscess edema are prime examples, involves disruption of the blood–brain barrier. As a result, iso-osmotic fluid and serum proteins enter the interstitial space from the bloodstream in response to a hydrostatic pressure difference. Therefore, in vasogenic brain edema, there is expansion of the interstitial space [20]. The resistance to flow of interstitial fluid is higher in gray matter (consisting of tangles of cell processes) compared with white matter (primarily consisting of aligned neuronal tracts), which explains why vasogenic edema fluid is found in the white matter on CT and MRI. Because the blood–brain barrier is open, vasogenic brain edema is associated with enhancement of the causative lesion (such as brain tumor) on CT or MRI scans after intravenous contrast administration.

*Hydrocephalic edema* refers to movement of CSF from the ventricles across the ependyma into the interstitial space in hydrocephalus. This type of brain edema has limited clinical significance other than providing evidence of hydrocephalus in situations where ventricular enlargement is unclear.

Despite the success of the Klatzo classification system, most clinical conditions consist of mixtures of different types of edema occurring with different time courses. For example, early cerebral ischemia produces cell swelling, but later on, when the capillary endothelium becomes damaged, the blood–brain barrier is disrupted, resulting in vasogenic-type swelling.

### Edema fluid elimination

**Fig. 2:**
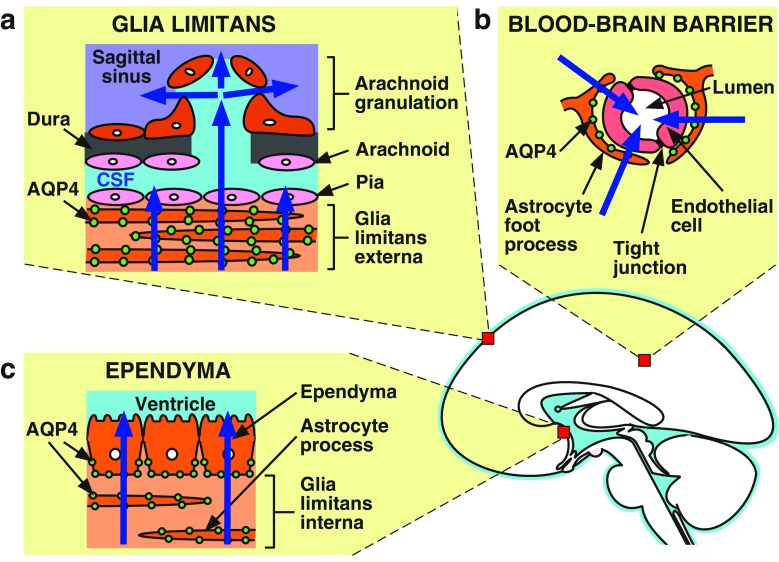
Routes of water exit from the brain in brain edema. In both cytotoxic and vasogenic types of brain edema, excess fluid is eliminated through aquaporin-4 (AQP4) rich barriers: **a** The glia limitans externa into the subarachnoid space; **b** the blood–brain barrier into the bloodstream; **c** the glia limitans interna and ependyma into the ventricles

The mechanisms of edema fluid clearance are less well understood than the mechanisms of edema fluid formation. In all brain edema types, excess fluid leaves the brain parenchyma along three different routes: across the blood–brain barrier into the bloodstream, across the ependyma into the ventricles, and across the glia-limiting membrane into the CSF in the subarachnoid space (Fig. 2). CSF eventually enters the arachnoid granulations and is cleared into the superior sagittal venous sinus.

The relative contributions of the three major routes of edema fluid elimination from the brain are not known. It has been suggested that vasogenic edema fluid is cleared primarily by bulk flow through the extracellular space and across the glia limitans into the CSF [21]. This idea is based on old experiments that measured clearance rates of inert dyes injected into brain extracellular space. The dyes were primarily eliminated into the CSF at equal rates independent of molecular weight, favoring a bulk-flow mechanism [21]. A major flaw with this interpretation is the unjustified assumption that dye and water efflux routes are identical. More recent data suggest that excess brain water elimination in vasogenic edema across the glia limitans involves a transcellular route, not bulk flow [22].

Death of neurons and glia in cytotoxic edema releases their intracellular contents into the extracellular space. Regulatory volume decrease is an interesting phenomenon whereby swollen cells release intracellular ions (mainly K^+^ and Cl^−^) and amino acids (such as taurine and glutamate) into the extracellular space [16]. This produces a decrease in cell size toward baseline. It is possible, then, that in cytotoxic edema, the excess water initially resides in the intracellular compartment but ultimately moves to the extracellular space due to cell death and regulatory volume decrease. Therefore, excess water elimination routes in cytotoxic edema may be the same as those in vasogenic edema.

## AQP4 and brain edema

### AQP4 in cytotoxic edema

**Fig. 3:**
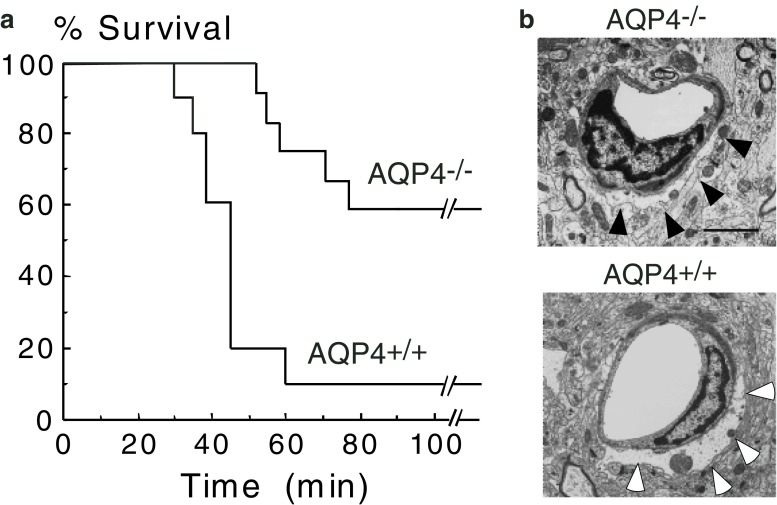
Aquaporin-4 (AQP4) null mice are protected from hyponatremic brain swelling. **a** Improved survival of AQP4-null (AQP4^−/−^) mice compared with wild-type (AQP4^+/+^) mice following water intoxication produced by injecting 0.2 ml/g body weight intraperitoneally. **b** Transmission electron micrograph showing edematous cerebral cortex at 30 min. Note the swollen astrocytic foot process in brains from AQP4^+/+^ (*black arrows*) and AQP4^−/−^ (*white arrows*) mice.* Scale bar* represents 3 μm

The first evidence that AQP4 plays a major role in cytotoxic brain edema came from studies with AQP4-null mice [23]. Intraperitoneal water injection causes profound hyponatremia to 105 mM within 5 min and subsequent death from brain swelling and increased intracranial pressure. Mortality after hyponatremia is markedly reduced in AQP4-null mice (Fig. 3). This protection is associated with reduced blood–brain barrier water permeability and reduced rate of water flow into the brain parenchyma. AQP4-null mice are also protected from other models of cytotoxic brain edema, including bacterial meningitis [23] and early focal cerebral ischemia [24]. AQP4 protein in plasma membranes is thought to be bound on an aggregate of intracellular proteins, including α-syntrophin [25]. Interestingly, α-syntrophin-null mice are also protected from cytotoxic brain edema produced by focal cerebral ischemia [26]. Taken together, these findings suggest that drugs that inhibit AQP4 expression or function might limit cytotoxic brain swelling in humans.

### AQP4 in vasogenic edema

An unexpected role for AQP4 in vasogenic brain edema was shown using three models of vasogenic brain edema in mice [22]. AQP4 null mice had more brain swelling compared with wild-type mice after cortical freeze injury and brain tumor implantation. The observation that intraparenchymal infusion is eliminated more slowly in AQP4-null mice compared with wild-type mice suggests that excess brain water elimination in vasogenic edema is defective after AQP4 deletion. Once the excess interstitial fluid (vasogenic edema) reaches the barriers between brain parenchyma and fluid compartments, it must be eliminated by a transcellular AQP4-dependent route. These findings suggest that AQP4 activators or upregulators, when available, may reduce vasogenic brain edema in humans.

### AQP4 in hydrocephalic edema

**Fig. 4:**
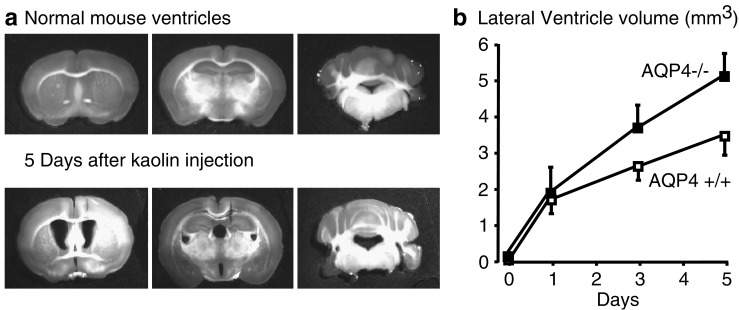
Aquaporin-4 (AQP4) deletion worsens ventricular enlargement in hydrocephalus. **a** Coronal brain sections showing ventricle size in a normal mouse (*top*) and in a mouse with obstructive hydrocephalus (*bottom*) produced by kaolin injection into the cisterna magna. **b** Lateral ventricle size in wild-type (AQP4^+/+^) and AQP4-null (AQP4^−/−^) mice following kaolin injection

Hydrocephalus is the accumulation of CSF with ventricular enlargement due to obstruction in CSF flow (obstructive or noncommunicating hydrocephalus) or impaired CSF absorption (communicating hydrocephalus). Causes of obstructive hydrocephalus can be congenital, e.g., aqueduct stenosis preventing CSF flow from the third to the fourth ventricle; or acquired, such as brainstem glioma or intraventricular hematoma. Communicating hydrocephalus is often associated with damage to the arachnoid granulations, as in meningitis. In a model of obstructive hydrocephalus produced by injecting kaolin in the cisterna magna of mice, AQP4-null mice had accelerated ventricular enlargement progression compared with wild-type mice (Fig. 4) [27]. Presumably, the reduced water permeability of the ependymal layer, subependymal astrocytes, and glia limitans produced by AQP4 deletion reduces the elimination rate of CSF across these borders into the subarachnoid space.

### Other functions of AQP4 in the brain

Apart from its role in brain edema, AQP4 was found to a play major role in cell migration [28–31] and neural excitability. Astrocyte migration is delayed in AQP4-null mice, and as a result, glial scarring is impaired [29, 31]. AQP4 is thought to accelerate astrocyte migration by facilitating transmembrane water flow, which accompanies the fast changes in cell shape that occur during migration. Several lines of evidence show reduced neural excitability in AQP4 deletion: AQP4-null mice have a higher seizure threshold and prolonged seizure duration than do wild-type mice [32, 33], and they have sensorineural deafness [34] and mild retinal impairment [35]. The mechanism by which AQP4 influences neural excitability is unknown but may involve interaction between AQP4 and an astrocytic potassium channel (Kir4.1) [36], or altered extracellular space size [37].

## Conclusion

In children, the main type of brain edema is cytotoxic due to fluid and electrolyte disturbances. However, vasogenic edema, as in brain tumors, is seen occasionally. AQP4 is important in the pathophysiology of cytotoxic and vasogenic brain edema, although it has opposing roles. Early on in cytotoxic edema, AQP4 facilitates edema fluid formation. In vasogenic brain edema, AQP4 increases the rate of edema fluid elimination. Therefore, AQP4 inhibitors are expected to protect the brain in cytotoxic edema, whereas AQP4 activators or upregulators would be required to facilitate the clearance of vasogenic brain edema. Unfortunately, there are no known activators or inhibitors of AQP4, but small-molecule discovery efforts are being directed toward that discovery.
